# The MINT Sprint 2.0: a Picture Naming Test for Detection of Naming Impairments in ΜCI Due or Not to AD, Greek Version

**DOI:** 10.1093/arclin/acaf106

**Published:** 2025-12-02

**Authors:** Aristi Alopoudi, Despina Moraitou, Thomas Tegos, Magda Tsolaki

**Affiliations:** Greek Association of Alzheimer Disease and Related Disorders, Petrou Sindika 13, Thessaloniki 54643, Greece; 2nd Department of Neurology, School of Medicine, Faculty of Health Sciences, Aristotle University of Thessaloniki, Thessaloniki 54124, Greece; Laboratory of Psychology, Department of Cognition, Brain and Behavior, School of Psychology, Faculty of Philosophy, Aristotle University of Thessaloniki (AUTh), Thessaloniki 54124, Greece; Laboratory of Neurodegenerative Diseases, Center of Interdisciplinary Research and Innovation (CIRI-AUTH), Balcan Center, Buildings A & B, Thessaloniki 57001, Greece; First Department of Neurology, Medical School, Aristotle University of Thessaloniki, Thessaloniki 54124, Greece; Greek Association of Alzheimer Disease and Related Disorders, Petrou Sindika 13, Thessaloniki 54643, Greece; 2nd Department of Neurology, School of Medicine, Faculty of Health Sciences, Aristotle University of Thessaloniki, Thessaloniki 54124, Greece; Laboratory of Neurodegenerative Diseases, Center for Interdisciplinary Research and Innovation (CIRI - AUTh), Thermi, Thessaloniki 57001, Greece

**Keywords:** Everyday functioning, Assessment, Alzheimer’s disease, Fluency (verbal/nonverbal), Language and language disorders, Mild cognitive impairment

## Abstract

**Objective:**

Naming impairments are among the earliest cognitive deficits in Alzheimer’s disease (ad), particularly affecting semantic memory and lexical retrieval. This study evaluated the diagnostic utility of the Greek adaptation of the MINT Sprint 2.0, a culturally tailored picture-naming test, in identifying naming deficits in preclinical and early ad.

**Method:**

A total of 170 Greek-speaking participants were classified into three diagnostic groups: (1) MCI biomarker positive (preclinical ad), (2) MCI biomarker negative, and (3) cognitively intact (CI) (biomarker negative). Participants completed the MINT Sprint 2.0, comprising naming accuracy (MINTFIRSTPASS) and response latency (MINTFIRSTPASSTIME). Multivariate and univariate ANOVAs were used to assess group differences.

**Results:**

Diagnosis significantly influenced performance across both measures, accounting for 26.5% of variance in accuracy and 52.7% in latency (*p* < .01). MANOVA revealed a large multivariate effect (Pillai’s Trace V = 0.674, *p* < .01). Post hoc tests confirmed that all groups differed significantly on both variables, with biomarker-positive individuals performing worse and slower than other groups. MINT Sprint 2.0 elicited significantly different performance across diagnostic groups, supporting its potential utility in detecting early AD-related changes and offering enhanced sensitivity to early lexical retrieval deficits and response speed. Strong correlations between MINT Sprint and 15-BNT scores supported convergent validity.

**Conclusion:**

The Greek MINT Sprint 2.0 is a valid and sensitive tool for detecting early ad-related naming impairments. Its cultural relevance and inclusion of response time make it superior to traditional naming tests, offering potential as a primary screening measure in Greek-speaking populations.

## INTRODUCTION

Neurodegenerative diseases (NDs) are a growing global health concern, driven by demographic shifts, lifestyle changes, and a rising number of affected individuals. These conditions significantly reduce patients’ quality of life, impose emotional and physical stress on caregivers, and create substantial financial burdens for healthcare systems (Alzheimer’s Association, 2022; Feigin et al., 2020; Hampel et al., 2018). Over 50 million people worldwide currently live with NDs—a figure projected to nearly triple by 2050 without effective prevention or treatment strategies (Nichols et al., 2022).

Alzheimer’s disease (ad), the most common neurodegenerative disorder, accounts for 60%–80% of dementia cases, marked by progressive memory loss and cognitive decline (GBD 2019 Dementia Forecasting Collaborators, 2022). It is characterized by the accumulation of amyloid-beta (Aβ) plaques and tau tangles, leading to synaptic dysfunction and neuronal death (Braak & Del Tredici, 2020). Recent studies suggest that soluble amyloid-beta oligomers (AβO), rather than plaques, play a more critical role in initiating synaptic injury in the preclinical stage, even before clinical symptoms or imaging abnormalities appear (Braak & Del Tredici, 2020; Grothe et al., 2021; Vergallo et al., 2019). Early detection of ad has been improved with biomarkers such as cerebrospinal fluid (CSF), Aβ42/Aβ40 ratios, phosphorylated tau, and advanced imaging techniques like amyloid and tau PET (Ardila, 2019; Bayat et al., 2024; Colvee-Martin et al., 2024; Vergallo et al., 2019). These methods align with the A-T-(N) framework from the National Institute on Aging–Alzheimer’s Association (NIA-AA), classifying individuals based on amyloid, tau, and neurodegeneration markers (Colvee-Martin et al., 2024; Hampel et al., 2018; Lin et al., 2021). Detecting changes at the preclinical or early prodromal stage is considered crucial for potentially altering disease progression (Hampel et al., 2018; Zhang et al., 2024; Yu et al., 2024).

The importance of cognitive testing in the early detection of ad cannot be overstated. Cognitive tests, particularly those that assess language abilities such as word retrieval and naming, are crucial for identifying subtle cognitive changes that precede overt clinical symptoms (Dumitrescu et al., 2025). These tests offer more than just diagnostic value (Smith, Ivanova & Fernandez, 2022); they serve as important tools for monitoring disease progression and assessing the effectiveness of interventions. Given the progressive nature of ad, early diagnosis can significantly impact treatment outcomes, allowing for early interventions that may slow disease progression, preserve cognitive function, and improve quality of life for affected individuals (Hampel et al., 2018; Zhang et al., 2024).

Furthermore, cognitive assessments are integral to differentiating between AD and other forms of dementia, particularly in the early stages when clinical differentiation can be challenging. For example, naming difficulties observed in ad differ from those seen in frontotemporal dementia or vascular dementia (Smith, Ivanova & Fernandez, 2022), enabling more accurate diagnosis based on patterns of cognitive decline and specific impairments (Ardila, 2019; Montine et al., 2019). Beyond diagnosis, cognitive tests help identify at-risk populations—individuals showing early cognitive decline without meeting criteria for mild cognitive impairment (MCI) or ad—thus facilitating targeted interventions and longitudinal monitoring (Petersen, 2016; Roberts & Knopman, 2013).

Moreover, the use of standardized cognitive tests provides a valuable framework for comparing patient outcomes across research studies, fostering a broader understanding of cognitive changes associated with ad and aiding the development of novel treatments and preventive strategies (García et al., 2021; Hampel et al., 2018). Therefore, incorporating naming and language assessments into clinical practice supports not only early detection but also ongoing cognitive health monitoring, which is vital in combating AD (Ardila, 2019; Zhang et al., 2024).

In parallel, one of the earliest cognitive domains affected in ad is language—especially word retrieval (Kim & Choi, 2024) and naming—making targeted naming assessments invaluable for early diagnosis (Ardila, 2019; Montine et al., 2019). While healthy aging may impair phonological retrieval, ad disrupts semantic processing and all stages of lexical access (Ardila, 2019; Montine et al., 2019). Patients often experience “tip-of-the-tongue” (TOT) states that persist despite phonological cues (Pournaghdali et al., 2025), and early episodic and semantic memory impairments often manifest as subtle language difficulties (Gagliardi & Vannini, 2022; Jiang & An, 2025).

MCI, a clinical syndrome marked by cognitive decline greater than expected for an individual’s age and education, but not severe enough to affect independence, carries a 10%–15% annual risk of progressing to ad (Busse et al., 2003; Gauthier et al., 2006; Petersen, 2004; Roberts & Knopman, 2013). Naming tests have become essential for detecting early cognitive decline associated with ad (Porsteinsson et al., 2021). Recent studies have validated these assessments as vital tools for identifying early signs of cognitive impairment in individuals at risk of ad dementia (García et al., 2021; Morkovina et al., 2024). For example, the Famous Faces Naming Test can distinguish between MCI patients likely to progress to ad and those who are not (Macoir et al., 2025). Similarly, the Addenbrooke’s Cognitive Examination-III (ACE-III) and the Montreal cognitive assessment (MoCA) have shown robust validity in detecting early ad symptoms, with MoCA demonstrating higher sensitivity than other screening tools for identifying MCI and early ad (Hsieh et al., 2013; Nasreddine et al., 2005; Poptsi et al., 2019). Additionally, the Alzheimer’s Disease Assessment Scale–Cognitive Subscale (ADAS-Cog), widely used to track cognitive decline, includes naming tasks critical for detecting early ad symptoms (Tsatali et al., 2024).

Recent reviews of neuropsychological assessments emphasize that naming deficits often appear in the preclinical stages of AD, highlighting their diagnostic importance in both clinical and research settings (Georgiou et al., 2022; Karalı et al., 2025; Martínez-Ferreiro, 2024; Soncu Büyükişcan, 2025; Stiver et al., 2022). This growing body of evidence underscores the critical role of naming tests in enabling early diagnosis, which is essential for timely intervention and potentially slowing disease progression (García-Gutiérrez et al., 2023; Zhang et al., 2024). These tests primarily assess object naming, which is significantly impaired in individuals with cognitive deficits (Georgiou et al., 2022). While traditional tests like the Boston Naming Test (BNT) (Kaplan, Goodglass, & Weintraub, 1983) have been widely used, their effectiveness may be compromised by cultural bias and limited item sets that do not adequately represent diverse populations (Shaikh et al., 2025). Other commonly employed tools, such as the Controlled Oral Word Association Test (COWAT) (Benton et al., 1983) and the Picture Naming Test (PNT) (Paplikar et al., 2022), may also face similar limitations regarding cultural sensitivity. To overcome these issues, newer naming assessments like the Multilingual Naming Test (MINT) (Gollan et al., 2024) and the Object and Action Naming Battery (OANB) (Bogka et al., 2003) have been developed, offering broader item sets and cross-cultural applicability (Gollan et al., 2024). Recent innovations, such as the Staggered Uneven Number (SUN) Test (Dahan et al., 2020) and the Mobile Universal Lexicon Evaluation System (MULES) (Zimmerer, Weiner & Varley, 2021; Cobbs et al., 2017), further improve sensitivity in detecting early ad (Hudson et al., 2022).

The MINT Sprint 2.0, designed to assess naming ability across multiple languages and cultures, incorporates a time-restricted design to stress lexical retrieval (Gollan et al., 2024). This innovative approach aims to improve early diagnosis and intervention strategies, contributing to better management of cognitive decline and quality of life improvements for at-risk individuals. The MINT Sprint 2.0 was selected over other contemporary naming assessments due to its unique combination of time-restricted administration, high-resolution color stimuli depicting objects and everyday scenes, and the capacity to measure both naming accuracy and retrieval speed. Its robust psychometric properties and recent validation studies provided a solid foundation for adaptation in Greek. Among available tools, it offers a more sensitive and comprehensive framework for detecting subtle lexical retrieval difficulties in adults.

The aim of the present study was to examine whether the Greek adaptation of MINT Sprint 2.0 is an appropriate tool to detect MCI and, primarily, to distinguish between MCI due to ad and MCI not due to ad.

Recent validation studies of the MINT Sprint 2.0 have demonstrated its effectiveness across diverse populations. Gollan et al. (2023) examined cognitively normal adults, individuals with MCI, and patients with ad across different linguistic and cultural backgrounds, including English-, Spanish-, and Chinese-speaking cohorts. The test showed high sensitivity and specificity in discriminating between healthy controls and those with MCI, with reported values exceeding 85%, thus confirming its diagnostic utility. Additionally, its time-pressured format was found to accentuate retrieval difficulties that are often subtle in early cognitive decline but may be missed by untimed assessments. This feature is particularly valuable given the heterogeneity of naming deficits in MCI and ad, which can vary with cultural and linguistic context (Gollan et al., 2023, 2024). These findings underscore the MINT Sprint 2.0’s adaptability and cross-cultural validity, making it a promising tool for international clinical settings.

The MINT Sprint 2.0 offers enhanced sensitivity to early lexical retrieval difficulties through its time-restricted format, capturing subtle processing speed impairments alongside naming accuracy. Its use of culturally neutral, high-quality images reduces bias and improves applicability across diverse populations. By measuring both accuracy and response latency, it provides a multidimensional assessment of language function, aiding earlier and more precise detection of cognitive decline and improving clinical decision-making in AD and MCI.

The hypotheses of the study were formulated as follows:

a) The Greek version of the Multilingual Naming Test 2.0 SPRINT (MINT 2.0 SPRINT) will be a reliable measure for assessing word retrieval and response times in Greek-speaking individuals, with performance significantly differing between cognitively intact individuals (CI) and those with MCI.
b) MCI patients with a positive Aβ biomarker (MCI biomarker positive) will show lower performance compared to those with a negative Aβ biomarker (MCI biomarker negative).
c) The MINT 2.0 SPRINT will show convergent validity, in terms of its comparison with the short version of the 15-BNT, indicating that both tests measure similar aspects of word retrieval ability, but the MINT 2.0 SPRINT provides additional insights into naming fluency and processing speed through its measurement of response times.

## MATERIALS AND METHODS

### Sample

The study’s design comprised three diagnostic groups: (a) persons diagnosed with MCI/Preclinical ad biomarker positive, (b) persons with MCI biomarker negative, and (c) CI (biomarker negative). All participants were volunteers recruited from the Greek Association of Alzheimer’s Disease and Related Disorders in Thessaloniki. All participants have been tested through lumbar puncture (LP), and we have the necessary biomarkers for the present study. As part of the study protocol, CI individuals also underwent LP.

LP is increasingly used in CI individuals to detect early ad pathology before symptoms appear. LP also helps exclude other neurological causes in at-risk individuals and is often required for participation in clinical trials targeting presymptomatic ad (García-Gutiérrez et al., 2023; Sewell et al., 2025). Monitoring CSF biomarkers over time can inform disease progression and treatment response, supporting personalized care (Chen et al., 2023; Wang et al., 2024).

The study sample comprised 170 native Greek speakers (Table 1). It consisted of 65 men and 105 women, with mean age = 67.05 years, SD = 8.64, ranging from 55 to 83 years**.** Participants had an average educational attainment with mean education = 11.83 years, SD = 3.37, with years of schooling varying from 6 to 15 years. Participants were divided into three diagnostic groups (Table 1): (a) MCI biomarker positive, classified according to the following DSM-5 criteria (American Psychiatric Association, 2022): cognitive decline in one or more domains (e.g., memory, executive function, language, attention) based on the concern of the individual, an informant, or clinician, and confirmed by objective evidence from neuropsychological testing. These deficits do not significantly impair independence in everyday activities but may require compensatory strategies. The condition must not occur exclusively during delirium or be better explained by another mental disorder. When attributed to Alzheimer’s, the decline is typically gradual and progressive, with evidence from genetic testing or clinical patterns confirming the etiology (Ebbesen et al., 2023) and exhibiting a positive biomarker for β-amyloid (Aβ; n = 57; 22 men and 35 women; M = 68.45 years, SD = 8.92; M = 11.70 years of education, ranging from 6 to 15 years, SD = 3.34); (b) people with MCI lacking evidence of Aβ pathology (negative biomarker; n = 57; 22 men and 35 women; M = 67.69 years, SD = 8.20; M = 11.83 years of education, ranging from 6 to 16 years, SD = 3.37); and (c) CI (n = 56; 21 men and 35 women; M = 64.97 years, SD = 8.57; M = 12.22 years of education, ranging from 6 to 15 years, SD = 3.63). Based on the MoCA scores, the MCI biomarker positive had a mean score of M = 22.50, SD = 2.20; the MCI biomarker negative had M = 23.03, SD = 2.37; and the CI group scored M = 29.03, SD = 0.65. The higher proportion of women in studies of MCI and ad is primarily due to demographic and biological factors. Women tend to live longer than men, and since age is the strongest risk factor for ad, their greater longevity leads to an overrepresentation of women in older populations (Zhu et al., 2021). Biologically, hormonal changes after menopause, particularly the decline in estrogen, may increase susceptibility to cognitive decline, as estrogen is thought to have neuroprotective effects (Bortz et al., 2022).

**Table 1:** Study sample demographic characteristics

**Characteristics**	**MCI Aβ + (n = 57)**	**ΜCI Αβ- (n = 57)**	**CI (n = 56) M (SD)**
Age	68.45 (8.92)	67.69 (8.20)	64.97 (8.57)
Gender	22/35	22/35	21/35
Education	11.70 (3.34)	11.83 (3.37)	12.22 (3.63)
MoCA	22.50 (2.20)	23.03 (2.37)	29.30 (0.65)

*Note. Aβ +* **=** (biomarker positive), *Aβ*- = (biomarker negative)

While MoCA was used as an initial screening tool, final group classification was based on clinical consensus diagnosis. The diagnostic classification of participants was confirmed through consensus by a team of specialized neurologists and neuropsychologists, based on a comprehensive evaluation that included neurological examination, detailed neuropsychological testing, review of medical history, neuroimaging (either computed tomography or magnetic resonance imaging), and relevant blood analyses (e.g., metabolic screening) (Weinstein et al., 2022).

Group comparisons were conducted to assess matching between diagnostic categories. Pearson’s chi-square test indicated that gender distribution did not differ significantly across the groups, χ^2^(2) = 0.038, *p* > .05. Similarly, one-way ANOVA revealed no significant differences in age among the groups, F(2, 170) = 2.561, *p* > .05. Regarding education, one-way ANOVA showed no significant differences in years of schooling, F(6, 855) = 0.659, *p* > .5. To further explore group-level differences, Scheffé post hoc tests were applied. These results suggest that educational attainment was comparable across all diagnostic groups.

MCI biomarker positive vs. MCI biomarker negative: I − J = 0.526, *p* > .05;

MCI biomarker positive vs. CI, biomarker negative: I − J = 0.656, *p* > .05;

MCI biomarker negative vs. CI, biomarker negative: I − J = 0.130, *p* > .05.

As expected, cognitive status—as measured by the MoCA—varied significantly across groups, F(2, 170) = 219.659, *p* < .05, with a large effect size (η^2^ = 0.725). This confirms substantial differences in global cognitive function consistent with diagnostic classification. Post hoc comparisons using the Scheffé method showed that individuals with MCI biomarker positive status scored significantly lower on the MoCA than CI (I–J = −6.794, *p <* .05). Similarly, those with MCI biomarker negative also scored significantly lower than CI (I–J = −6.268, *p <* .05). However, the comparison between MCI biomarker positive and MCI biomarker negative groups showed no significant difference in scores (I–J = 0.526, *p* > .05). This indicates that cognitive performance does not differ notably between the two MCI subgroups. The overall analysis supports that both MCI groups performed lower on the MoCA compared to typical controls, regardless of biomarker status.

Exclusion criteria for all groups encompassed psychiatric history, recent major health issues such as history of TBI, stroke, seizures/epilepsy, drug use, vitamin deficiency, metabolic dysfunction, untreated sleep apnea, and medications affecting cognitive function. The extended exclusion criteria were designed to ensure, as fully as possible, that both the CI group and the MCI groups were free from other factors that could potentially impact cognitive functioning. A comprehensive neuropsychological assessment was conducted for all participants (Georgopoulou et al., 2023). To exclude depressive and anxiety disorders, participants were assessed using the Geriatric depression scale-15 item version (GDS-15) and Beck depression inventory (BDI) (Beck et al., 1961), the Short Anxiety Screening Test (SAST) (Grammatikopoulos et al., 2010) and the Beck anxiety inventory (BAI) (Beck et al., 1988). Participants scoring above the following cutoffs were excluded to rule out depressive and anxiety disorders: GDS ≥ 5, BDI ≥ 14, SAST ≥24, and BAI ≥ 16. The Neuropsychiatric Inventory was administered to screen for neuropsychiatric symptoms that could affect cognition (Cummings, 2020). Cognitive status was evaluated using the Mini Mental State Examination (Folstein et al., 1975; Fountoulakis et al., 2000) and the MoCA (Nasreddine et al., 2005; Poptsi et al., 2019). The Functional Cognitive Assessment (Kounti et al., 2006) measured participants’ capacity to manage six Activities of Daily Living. Additional standardized tests assessed a range of cognitive functions, including memory, language, executive function, and attention. Participants’ disease progression was categorized according to the Global Deterioration Scale (Reisberg et al., 2019). Lastly, cognitive complaints among CI and MCI groups were evaluated using the first three questions of the subjective cognitive decline questionnaire (SCD-Q) (Rami et al., 2014).

The inclusion criteria were as follows: (a) a diagnosis of MCI according to the Diagnostic and Statistical Manual of Mental Disorders (5th ed.; DSM-5), (b) a Mini-Mental State Examination (MMSE) total score of ≤24/30, (c) classification at stage 3 of the disease based on the Global Deterioration Scale, and (d) cognitive performance at least 1.5 standard deviations (SD) below the normative mean for age and education in at least one cognitive domain, as determined by the administered neuropsychological assessments. Classification into the MCI biomarker positive or MCI biomarker negative groups was based on biomarker status (Poptsi et al., 2023; Jack et al., 2024).

For the CI group the inclusion criteria were as follows: (a) participants were required to be over the age of 50, (b) at least 6 years of formal education (c) participants needed to show no subjective cognitive complaints, verified by negative answers to the first three items of the SCD-Q, and to be (d) only those at stage 1 on the GDS), indicating no observable cognitive impairment or minimal initial decline.

### Tools

All groups were evaluated using both the Greek version of the MINT Sprint 2.0 and the short version of the Greek 15-BNT (Patricacou et al., 2007).

Α. The MINT and its updated version, MINT Sprint 2.0, are tools specifically designed to evaluate lexical retrieval and confrontational naming abilities. Unlike traditional naming tests, such as the BNT (Patricacou et al., 2007), the MINT prioritizes cultural neutrality by including items that are easily recognizable not only across monolingual individuals but also various linguistic and cultural backgrounds. This feature makes it especially valuable for assessing multilingual individuals, reducing the potential biases related to language, education, or cultural familiarity.

The MINT Sprint 2.0 is a shortened and time-efficient version of the original MINT. It includes a carefully selected subset of stimuli that balance difficulty and word frequency, ensuring that the test remains sensitive to a wide range of cognitive abilities while requiring less time for administration. Participants are shown images of common objects, actions, or items and asked to name them. The test records naming accuracy, and the total time taken to name all items. From these data, key metrics such as the efficiency score and total naming time are calculated, offering a comprehensive view of word-retrieval performance. The MINT Sprint 2.0 is particularly effective in detecting early lexical retrieval deficits associated with preclinical ad and other language-related cognitive impairments. It has been validated for use in both clinical and research contexts, making it a versatile tool for neuropsychological assessment. The test’s cultural inclusivity and diagnostic precision are complemented by its practicality in time-constrained settings, as it allows for the efficient evaluation of naming abilities without compromising diagnostic accuracy. By combining cultural fairness with time efficiency, the MINT Sprint 2.0 addresses the limitations of traditional naming tests while maintaining sensitivity to cognitive decline. Its metrics, including the efficiency score and total naming time, provide robust insights into lexical retrieval and fluency, making it an excellent tool for studies focused on early cognitive changes.

MINT Sprint 2.0 test utilizes a laminated card measuring 17 × 13 inches. This card features eight rows, each containing 10 color photographs of various objects, with each photo measuring approximately 1 to 1.5 square inches. The MINT Sprint 2.0 test includes 80 items. All images are presented simultaneously, and the more challenging items, intended to provoke TOT states, are positioned in the lower rows. The same rule exists in the Greek version of the test. Some items in the original MINT Sprint 2.0 were modified to mitigate issues with overly guessable images which could compromise individuals’ accuracy in naming objects (Stasenko et al., 2019).

For the Greek adaptation of MINT Sprint 2.0, three images were modified to enhance the test’s validity for Greek-speaking participants. Based on pilot data from 10 native Greek speakers, the following substitutions were implemented: “gauge/barometer” was replaced with “accordion,” “mortar/pestle” with “compass,” and “bellows” with “horseshoe.” These changes were made following multiple trial rounds, as the new terms are more commonly used in Greek and are widely recognized across varying levels of educational background (Gollan et al., 2023).

To instill a sense of urgency, participants have 3 min to name as many images as possible, starting from the top-left corner and quickly progressing through each row. The examiner encourages participants with prompts such as “keep going,” advising them to limit their response time to three to 4 s per image. They are allowed to revisit any images they initially skipped. While there is no strict time limit, most participants complete their initial pass through the grid in less than 3 min. The instructions provided are: “I will show you eight rows of pictures. Please try to name each image in Greek as quickly as possible without making mistakes. Start in the top-left corner. If you encounter an image you cannot name or remember, say ‘don’t know’ and move on. You can come back later if you remember the name. You have 3 minutes to name as many pictures as possible.”

The duration of the test is recorded. The time spent naming pictures is a critical variable that records the amount of time a participant spends identifying and naming each picture presented during the task. This is typically tracked in seconds or minutes and is crucial for understanding the speed of cognitive processing during recall tasks. The score of the first pass, on the other hand, refers to the accuracy of a participant’s first attempt at naming the pictures without any assistance or corrective feedback. This score provides insights into a participant’s immediate recall ability. The first pass score is especially valuable in assessing how well the participant can recall or recognize visual stimuli from memory without prompts or interventions, which is often a key measure in cognitive assessments (Bruyer & Brysbaert, 2011; Gollan et al., 2024). The MINTFIRSTPASS variable quantified naming accuracy by recording the total number of correct responses produced during the initial attempt to name items, without prompts or repetition, thus serving as a measure of spontaneous word retrieval. The MINTFIRSTPASSTIME variable captured the total duration of time spent naming pictures, reflecting processing speed and the cognitive load associated with lexical access.

B. The 15-BNT is a shortened version of the original BNT (Abeare et al., 2022; Patricacou et al., 2007), widely used to assess confrontational word retrieval and naming ability. It is particularly sensitive to language deficits often observed in neurodegenerative conditions such as MCI and ad. The Greek adaptation of the 15-BNT has been standardized and validated for use in Greek-speaking populations, ensuring cultural and linguistic appropriateness. It includes 15 black-and-white line drawings of increasing difficulty, and the participant is asked to name each object. Performance on the 15-BNT can offer insight into lexical retrieval processes and may be affected by factors such as age, education, and underlying cognitive decline.

### Procedure

The assessment procedures were conducted at the offices of Alzheimer Hellas in Thessaloniki. Each participant attended individually by scheduled appointment during morning hours. Upon arrival, participants were informed about the study protocol and provided written consent prior to test administration. The entire process lasted approximately half an hour per participant. No difficulties or adverse events were encountered during the assessments.

### Data Analysis

To test the proposed hypotheses, data were analyzed using SPSS version 29.0 with a significance level set at α = 0.05 for all tests (IBM Corp., 2022).

The present study investigated group differences (MANOVA) in lexical retrieval performance using two dependent measures derived from the MINT SPRINT 2.0: first pass accuracy (MINTFIRSTPASS) and response time (MINTFIRSTPASSTIME). The independent variable was diagnostic group, which included three levels: MCI biomarker positive, MCI biomarker negative, and CI.

To assess the convergent validity of the MINT 2.0 SPRINT, Pearson’s correlation analysis (Cohen et al., 2003) was performed to examine the relationship between MINTFIRSTPASS score and the score on the short version of the 15-BNT (Abeare et al., 2022).

## RESULTS

### Diagnostic Group Differences in the MIND Sprint 2.0.

MANOVA was conducted to examine the effects of Diagnosis on MINTFIRSTPASS and MINTFIRSTPASSTIME variables. Pillai’s Trace revealed a significant multivariate effect of Diagnosis, V = 0.674, F(6, 332) = 28.105, *p <* .05, η^2^ = 0.337. The medium effect size indicates a strong impact of diagnosis on Mint Sprint 2.0 performance, across both the accuracy (MINTFIRSTPASS) and response time (MINTFIRSTPASSTIME) measures.

Following the MANOVA, separate univariate ANOVAs were conducted for each of the dependent variables. The first ANOVA tested the effect of Diagnosis on MINTFIRSTPASS, which measures first-pass accuracy. The results revealed a significant effect of Diagnosis, F(2, 167) = 30.059, *p* < .05, η^2^ = 0.265. This suggests that the accuracy in the MINT Sprint 2.0 test differs significantly in the three diagnostic groups. A subsequent ANOVA tested the effect of Diagnosis on MINTFIRSTPASSTIME, which measures the response time. The analysis also found a significant effect of Diagnosis, F(2, 167) = 92.993, *p* < .05, η^2^ = 0.527. The larger effect size for response time indicates that diagnosis has a stronger influence on the MINTFIRSTPASSTIME than on the MINTFIRSTPASS.

**Fig. 1:**
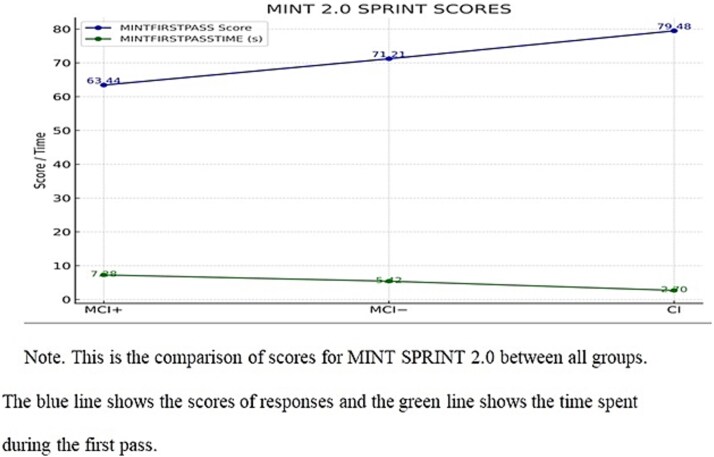
*Comparison of MINT FIRSTPASS scores and MINT FIRSTPASS time between groups*.

The Scheffe post hoc test was employed to examine the differences between the Diagnostic groups (MCI biomarker positive, MCI biomarker negative, and CI for both the MINTFIRSTPASS and MINTFIRSTPASSTIME. For MINTFIRSTPASS, the analysis revealed significant differences between all pairs of groups. Specifically, MCI biomarker positive had a significantly lower score than MCI biomarker negative (I-J = −8.47, *p* < .05) and CI (I-J = −15.77, *p* < .05). Additionally, MCI biomarker negative had a significantly lower score than CI (I-J = −7.30, *p* < .05). These findings suggest that CI achieved the highest MINTFIRSTPASS score, followed by MCI biomarker negative, with MCI biomarker positive to display the lowest scores (Fig. 1).

Regarding MINTFIRSTPASSTIME, the test also revealed significant differences across all comparisons. MCI biomarker positive required significantly more time than MCI biomarker negative (I-J = 1.86, *p* < .05), and CI (I-J = 4.59, *p* < .05), while MCI biomarker positive took significantly more time than CI (I-J = 2.72, *p* < .05). These results indicate that CI took the least time to complete the task, followed by MCI biomarker negative, and MCI biomarker positive required the most time.

### Convergent Validity of the MIND Sprint 2.0

There was a medium positive correlation between MINTFIRSTPASS and 15-BNT (r = 0.661, *p* < .05). There also was a relatively high negative correlation between MINTFIRSTPASSTIME and 15-BNT(r = −0.719, *p* < .05).

## DISCUSSION

The aim of the present study was to examine the diagnostic validity and clinical utility of the Greek version of the MINT 2.0 SPRINT as a sensitive and time-efficient tool for assessing lexical retrieval and processing speed in individuals across the cognitive spectrum. Specifically, the study explored its ability to differentiate between CI and two groups with MCI (biomarker positive and biomarker negative), with a further emphasis on the role of Aβ biomarker positivity in modulating test performance. In contrast to previous studies that primarily evaluated naming accuracy alone, the current approach incorporated response time as a core metric, allowing for a more comprehensive evaluation of cognitive-linguistic performance. In addition, this study directly addressed the convergent validity of the MINT 2.0 SPRINT with the 15-BNT, while also analyzing the influence of biomarker status—a factor not systematically explored in earlier Greek-language validation efforts.

### Diagnostic Sensitivity of the Greek MINT 2.0 SPRINT

The primary hypothesis of the study proposed that the Greek adaptation of the MINT 2.0 SPRINT would demonstrate diagnostic sensitivity in assessing lexical retrieval and processing speed among Greek-speaking individuals. The results lend robust support to this hypothesis, affirming the tool’s sensitivity to early cognitive impairment.

Multivariate analyses revealed that diagnostic classification significantly influenced performance on the MINT 2.0 SPRINT, both in terms of naming accuracy and response time. Specifically, individuals diagnosed with MCI and positive biomarkers for ad (i.e., amyloid-beta positivity) performed significantly worse than both MCI biomarker negative participants and CI. These findings are congruent with recent literature indicating that amyloid-positive MCI is often accompanied by prominent deficits in lexical access and semantic processing (García-Gutiérrez et al., 2023; Mueller, 2023). There are several pathophysiological pathways hypothesized to be responsible for these deficiencies. Particularly in the temporoparietal cortices and the anterior temporal lobe, areas crucial for the storage and retrieval of semantic information, amyloid-beta oligomers promote synaptotoxic consequences by interfering with long-term potentiation and causing dendritic spine loss (Zhang et al., 2022). Large-scale networks such as the default mode network and the semantic control network are less effective as a result of this synaptic dysfunction. Additionally, amyloid buildup in the precuneus and posterior cingulate cortex reduces the integration of conceptual knowledge during word retrieval tasks by compromising functional connection between the anterior temporal pole and left angular gyrus (Ingala et al., 2021). Controlled selection of competing lexical candidates is supported by neuroimaging investigations that have also shown hypometabolism and altered oscillatory dynamics in the left inferior frontal lobe (Chiang et al., 2024; Dimitrescy et al., 2025). Simultaneously, amyloid-related neuroinflammation exacerbates synaptic loss by activating microglia and encouraging the local production of proinflammatory cytokines, which further adds to network breakdown. When combined, these processes lead to a decreased ability to access stored semantic knowledge efficiently and a greater need for compensatory or circumlocutory language production techniques (Lemprière, 2023; Cai et al., 2022).

Crucially, people with MCI biomarker negative also perform worse on tests of language and semantic memory. This observation could be explained by a number of ways. First, gradual neuronal deterioration, particularly in medial temporal and frontoparietal regions crucial for language, can be caused by non-ad diseases such as primary age-related tauopathy (PART), hippocampal sclerosis, and cerebrovascular illness (Chang et al., 2022; Kitaigorodsky et al., 2021; Teylan et al., 2020). Second, regardless of amyloid load, age-related synapse loss and neuroinflammation may reduce functional connection within executive and semantic control networks (Al-Ezzi et al., 2024). Third, a variety of etiologies, such as Lewy body disease or TDP-43 proteinopathies, which might interfere with semantic retrieval processes, are probably responsible for certain MCI biomarker negative individuals (Bayram, Shan, and Cummings, 2019). Lastly, it has been demonstrated that neuronal network efficiency and cognitive reserve are adversely affected by psychological and physiological factors, including metabolic dysregulation, vascular risk factors, and chronic stress. These factors contribute to minor but quantifiable reductions in language ability (Jiang et al., 2025; McAleese et al., 2017; Szymkowicz et al., 2023). The combination of reduced accuracy and increased response time in this group underscores the test’s ability to capture both the efficiency and effectiveness of language production under cognitive load.

Recent neurocognitive models emphasize that lexical retrieval is among the earliest linguistic functions to decline in the prodromal stages of ad (Hamrick et al., 2023; Zhang et al., 2024). Such impairments often precede more global cognitive deterioration and are linked to early disruption in left-hemispheric temporal and parietal networks (Yoon et al., 2023). In this context, the MINT 2.0 SPRINT serves as a valuable diagnostic marker. Importantly, our findings align with a growing body of research advocating for composite metrics—such as accuracy—in cognitive screening tools. These metrics have been shown to improve diagnostic specificity in identifying MCI and early ad, especially when integrated with biomarker data (Ferasat et al., 2025; Grothe et al., 2021). In sum, the current findings validate the diagnostic relevance and linguistic appropriateness of the Greek MINT 2.0 SPRINT.

### The Impact of Biomarker Positive on Naming Accuracy and Processing Speed

The influence of biomarker positivity on language performance should therefore be viewed as a crucial extension of the diagnostic sensitivity results. Amyloid-related disruption of temporoparietal and default mode networks heightens vulnerability in tasks requiring rapid lexical access, making confrontation naming particularly sensitive to prodromal ad (Yoon et al., 2023; Gollan et al., 2024). By demonstrating that biomarker-positive MCI participants show greater impairments in accuracy and processing speed, our findings confirm the added value of integrating biological markers with behavioral measures. This multimodal approach strengthens the ecological and diagnostic validity of the Greek MINT 2.0 SPRINT and underscores its potential for early detection and individualized intervention strategies.

### Convergent Validity of the MINT 2.0 SPRINT with Established Language Measures

The third hypothesis posited that the MINT 2.0 SPRINT would demonstrate convergent validity by correlating with other established language assessments, such as the short form of the 15-BNT. The findings confirmed this hypothesis, revealing significant associations between MINT 2.0 SPRINT performance and both naming accuracy and broader cognitive-linguistic function.

These results align with previous literature suggesting that confrontation naming tasks are effective in capturing lexical retrieval abilities, particularly in populations with cognitive impairment (Kim & Choi, 2024; Stasenko et al., 2019). The significant associations observed in this study indicate that individuals who perform well on the MINT 2.0 SPRINT also tend to score higher on traditional language assessments. This supports the MINT 2.0 SPRINT’s validity as a measure of core language functions implicated in aging and early neurodegenerative processes.

Importantly, the MINT 2.0 SPRINT offers unique advantages over traditional instruments like the BNT. Its inclusion of a time-pressured component allows for a more nuanced analysis of lexical access speed, an area increasingly recognized as sensitive to early cognitive decline (Hamrick et al., 2023; Yoon et al., 2023). While the BNT remains a widely used tool, its untimed format may overlook subtle deficits in processing efficiency that are more readily captured through response latency measures.

Furthermore, the test’s cross-linguistic adaptability enhances its utility in clinical contexts. Unlike the 15-BNT, which has been criticized for cultural and linguistic biases, the MINT was first developed with multilingual and multicultural applicability in mind (Gollan et al., 2024). The present study demonstrates that the Greek version of the MINT 2.0 SPRINT maintains this adaptability for Greek speakers.

## LIMITATIONS

While the findings of this study are promising, several limitations should be acknowledged. First, the sample size was relatively small, and future studies should include larger sample to enhance the generalizability of the results. Additionally, the study relied on cross-sectional data, meaning that we cannot draw conclusions about the progression of cognitive decline over time. Longitudinal studies are needed to assess how performance on the MINT Sprint 2.0 changes as individuals move from MCI to more severe stages of dementia. The study did not evaluate the tool’s ability to distinguish between people with SCD and CI, which is arguably its most significant drawback. The requirement for tools that can identify neurodegenerative illness in its earliest, pre-MCI stages rather than just concentrating on more severe impairment makes this distinction crucial. Future research should focus on creating and validating metrics that are sensitive to minute alterations in semantic processing and lexical retrieval in SCD populations.

## FUTURE RESEARCH

Future research should explore the longitudinal efficacy of the MINT Sprint 2.0 in tracking cognitive decline over time, particularly in individuals with MCI. Additionally, studies could investigate how performance on the MINT Sprint 2.0 correlates with biomarkers of neurodegeneration, such as amyloid PET scans, to better understand the biological underpinnings of cognitive impairment. Further, examining the impact of interventions on MINT Sprint 2.0 performance could provide valuable insights into the effectiveness of treatments for MCI and early-stage dementia.

## CONCLUSION

The Greek version of the MINT 2.0 SPRINT was assessed to distinguish between the cognitive performance of MCI biomarker positive, MCI biomarker negative and CI. With notable variations in the performance seen between groups, it was discovered that the instrument is sensitive and dependable for evaluating word retrieval and response times. The MINT 2.0 SPRINT’s convergent validity with the 15-BNT was confirmed by the study. According to the results, the MINT 2.0 SPRINT may prove to be a valuable instrument for the early identification and distinction of cognitive deterioration, especially in MCI and ad.

## Data Availability

Data generated during this study are available from the corresponding author on reasonable request.

## References

[ref1] Abeare, K., Cutler, L., An, K. Y., Razvi, P., Holcomb, M., & Erdodi, L. A. (2022). BNT-15: Revised performance validity Cutoffs and proposed clinical classification ranges. Cognitive and behavioral neurology : official journal of the Society for Behavioral and Cognitive Neurology, 35, 155–168. 10.1097/WNN.0000000000000304. 35507449

[ref2] Al-Ezzi, A., Arechavala, R. J., Butler, R., Nolty, A., Kang, J. J., Shimojo, S., et al. (2024). Disrupted brain functional connectivity as early signature in cognitively healthy individuals with pathological CSF amyloid/tau. Communications Biology, 7, 1037. 10.1038/s42003-024-06673-w. 39179782 PMC11344156

[ref3] Alzheimer’s Association (2022). 2022 Alzheimer’s disease facts and figures. Alzheimers Dement, 18, 700–789. 10.1002/alz.12638. 35289055

[ref4] American Psychiatric Association (2022). DSM-5-TR neurocognitive disorders supplement. https://psychiatryonline.org/pb-assets/dsm/update/DSM-5-TR_Neurocognitive-Disorders-Supplement_2022_APA_Publishing.pdf.

[ref5] Ardila, A. (2019). Executive functions brain functional system. In Ardila, A., Fatima, S., & Rosselli, M. (Eds.), Dysexecutive syndromes: Clinical and experimental perspectives (pp. 29–41). Switzerland AG: Springer Nature. 10.1007/978-3-030-25077-5_2.

[ref6] Bayat, S., Sanati, M., Mohammad-Panahi, M., Khodadadi, A., Ghasimi, M., Rezaee, S., et al. (2024). Language abnormalities in Alzheimer's disease indicate reduced informativeness. Ann Clin Transl Neurol, 11, 2946–2957. 10.1002/acn3.52205. 39291771 PMC11572728

[ref6a] Bayram, E., Shan, G., & Cummings, J. L. (2019) Associations between Comorbid TDP-43, Lewy Body Pathology, and Neuropsychiatric Symptoms in Alzheimer’s Disease. Journal of Alzheimer’s Disease, 69, 953–961. 10.3233/jad-181285. PMC6597983 31127776

[ref7] Beck, A. T., Epstein, N., Brown, G., & Steer, R. (1988). An Inventory for Measuring Clinical Anxiety: Psychometric Properties. Journal of Consulting and Clinical Psychology, 56, 893–897. 10.1037/t02025-000. 3204199

[ref8] Beck, A. T., Ward, C. H., Mendelson, M., Mock, J., & Erbaugh, J. (1961). An inventory for measuring depression. Arch Gen Psychiatry, 4, 561–571. 10.1001/archpsyc.1961.01710120031004. 13688369

[ref9] Benton, A. L., Hamsher, D. S. K., & Sivan, A. B. (1983). Controlled oral word association test (COWAT) [database record]. APA PsycTests. 10.1037/t10132-000.

[ref10] Bogka, N., Masterson, J., Druks, J., Fragkioudaki, M., Chatziprokopiou, E.-S., & Economou, K. (2003). Object and action picture naming in English and Greek. Eur J Cogn Psychol, 15, 371–403. 10.1080/09541440303607.

[ref11] Bortz, J., Klatt, K. C., & Wallace, T. C. (2022). Perspective: Estrogen and the risk of cognitive decline: A missing choline(rgic) link? Advances in nutrition (Bethesda, Md), 13, 376–387. 10.1093/advances/nmab145. 34849527 PMC8970832

[ref12] Braak, H., & Del Tredici, K. (2020). Spreading of tau pathology in sporadic Alzheimer’s disease along cortico-cortical top-down connections. Cereb Cortex, 28, 3372–3384. 10.1093/cercor/bhy152. PMC6095209 29982389

[ref13] Bruyer, R., & Brysbaert, M. (2011). Combining speed and accuracy in cognitive psychology: Is the inverse efficiency score (IES) a better dependent variable than the mean reaction time (RT) and the percentage of errors (PE)? Psychologica Belgica, 51, 5–13. 10.5334/pb-51-1-5.

[ref14] Busse, A., Bischkopf, J., Riedel-Heller, S. G., Angermeyer, M. C., & Leipzig Longitudinal Study of the Aged LEILA75+ (2003). Mild cognitive impairment: Prevalence and predictive validity according to current approaches. Acta Neurol Scand, 108, 71–81. 10.1034/j.1600-0404.2003.00118.x. 12859282

[ref15] Cai, Y., Liu, J., Wang, B., Sun, M., & Yang, H. (2022). Microglia in the neuroinflammatory pathogenesis of Alzheimer's disease and related therapeutic targets. Front Immunol, 13, 856376. 10.3389/fimmu.2022.856376. 35558075 PMC9086828

[ref16] Chang, H. I., Hsu, S. W., Kao, Z. K., Lee, C. C., Huang, S. H., Lin, C. H., et al. (2022). Impact of amyloid pathology in mild cognitive impairment subjects: The longitudinal cognition and surface morphometry data. Int J Mol Sci, 23, 14635. 10.3390/ijms232314635. 36498962 PMC9738566

[ref16a] Chen, C. D., Ponisio, M. R., Lang, J. A., Flores, S., Schindler, S. E., Fagan, A. M., Morris, J. C., & Benzinger, T. L. S. (2023) Comparing Tau PET Visual Interpretation with Tau PET Quantification, Cerebrospinal Fluid Biomarkers, and Longitudinal Clinical Assessment. Journal of Alzheimer’s Disease, 93, 765–777. 10.3233/jad-230032. PMC10200228 37092225

[ref17] Chiang, H. S., Mudar, R. A., Dugas, C. S., Motes, M. A., Kraut, M. A., & Hart, J., Jr. (2024). A modified neural circuit framework for semantic memory retrieval with implications for circuit modulation to treat verbal retrieval deficits. Brain Behav, 14, e3490. 10.1002/brb3.3490. 38680077 PMC11056716

[ref18] Cobbs, L., Hasanaj, L., Amorapanth, P., Rizzo, J. R., Nolan, R., Serrano, L., et al. (2017). Mobile universal lexicon evaluation system (MULES) test: A new measure of rapid picture naming for concussion. J Neurol Sci, 372, 393–398. 10.1016/j.jns.2016.10.044. 27856005 PMC5480375

[ref19] Cohen, J., Cohen, P., West, S. G., & Aiken, L. S. (2003). Applied multiple regression/correlation analysis for the behavioral sciences (3rd ed.). New York, NY: Routledge.

[ref20] Colvee-Martin, H., Parra, J. R., Gonzalez, G. A., Barker, W., & Duara, R. (2024). Neuropathology, neuroimaging, and fluid biomarkers in Alzheimer's disease. Diagnostics (Basel, Switzerland), 14, 704. 10.3390/diagnostics14070704. 38611617 PMC11012058

[ref21] IBM Corp (2022). IBM SPSS statistics for windows (version 29.0) [computer software]. IBM Corp.

[ref22] Cummings, J. (2020). The neuropsychiatric inventory: Development and applications. J Geriatr Psychiatry Neurol, 33, 73–84. 10.1177/0891988719882102. 32013737 PMC8505128

[ref23] Dahan, N., Moehringer, N., Hasanaj, L., Serrano, L., Joseph, B., Wu, S., et al. (2020). The SUN test of vision: Investigation in healthy volunteers and comparison to the mobile universal lexicon evaluation system (MULES). J Neurol Sci, 415, 116953. 10.1016/j.jns.2020.116953. 32554181

[ref24] Dumitrescu, A. M., Coolen, T., Wens, V., Rovai, A., Trotta, N., Goldman, S., et al. (2025). Investigating the Spatio-temporal signatures of language control-related brain synchronization processes. Hum Brain Mapp, 46, e70109. 10.1002/hbm.70109. 39835602 PMC11747998

[ref25] Ebbesen, S. U., Høgh, P., & Zibrandtsen, I. (2023). Plasma aβ biomarker for early diagnosis and prognosis of Alzheimer's disease - a systematic review. Dan Med J, 70, A07220446. 37341353

[ref26] Egbuchulem, K. I. (2024). The Karl Pearson's Chi-Square: A medical research libero, and a versatile test statistic: An editorial. Annals of Ibadan postgraduate medicine, 22, 5–8. PMC11848378 40007702

[ref27] Feigin, V. L., Vos, T., Alahdab, F., Amit, A. M. L., Bannick, M. S., Bekuma, T. T., et al. (2020). Burden of neurological disorders across the US from 1990–2017: A global burden of disease study. JAMA Neurol, 77, 683–1290. 10.1001/jamaneurol.2020.1127. 33136137 PMC7607495

[ref27a] Ferasat, M., Ghoochani, B. Z., Kaveh, M. H., Caycho-Rodriguez, T., & Asadollahi, A. (2025). Correction: Comparative analysis of five diagnostic tools in detecting mild cognitive impairment in older adults. Middle East Current Psychiatry, 32. 10.1186/s43045-025-00544-8.

[ref28] Folstein, M. F., Folstein, S. E., & McHugh, P. R. (1975). Mini-mental state: A practical method for grading the cognitive state of patients for the clinician. J Psychiatr Res, 12, 189–198. 10.1016/0022-3956(75)90026-6. 1202204

[ref29] Fountoulakis, K. N., Tsolaki, M., Chantzi, H., & Kazis, A. (2000). Mini mental state examination (MMSE): A validation study in Greece. Am J Alzheimers Dis Other Dement, 15, 342–345. 10.1177/153331750001500604.

[ref30] Gagliardi, G., & Vannini, P. (2022). Episodic memory impairment mediates the loss of awareness in mild cognitive impairment. Front Aging Neurosci, 13, 802501. 10.3389/fnagi.2021.802501. 35126092 PMC8814670

[ref31] García, S., Cuetos, F., Novelli, A., & Martínez, C. (2021). Famous faces naming test predicts conversion from mild cognitive impairment to Alzheimer's disease. Acta Neurol Belg, 121, 1721–1727. 10.1007/s13760-020-01483-3. 32886274

[ref32] García-Gutiérrez, F., Marquié, M., Muñoz, N., Alegret, M., Cano, A., de Rojas, I., et al. (2023). Harnessing acoustic speech parameters to decipher amyloid status in individuals with mild cognitive impairment. Front Neurosci, 17, 1221401. 10.3389/fnins.2023.1221401. 37746151 PMC10512723

[ref33] Gauthier, S., Reisberg, B., Zaudig, M., Petersen, R. C., Ritchie, K., Broich, K., et al. (2006). Mild cognitive impairment. Lancet (London, England), 367, 1262–1270. 10.1016/S0140-6736(06)68542-5. 16631882

[ref34] GBD 2019 Dementia Forecasting Collaborators (2022). Estimation of the global prevalence of dementia in 2019 and forecasted prevalence in 2050: An analysis for the global burden of disease study 2019. Lancet Public Health, 7, e105–e125. 10.1016/S2468-2667(21)00249-8. 34998485 PMC8810394

[ref35] Georgiou, E. Z., Prapiadou, S., Thomopoulos, V., Skondra M., Charalampopoulou M., Pachi A., Anagnostopoulou Α., Vorvolakos T., Perneczky R., Politis A., Alexopoulos P., & others. (2022). Naming ability assessment in neurocognitive disorders: A clinician’s perspective. BMC Psychiatry, 22, 837. 10.1186/s12888-022-04486-x, 0 36585667 PMC9801565

[ref36] Georgopoulou, E. N., Nousia, A., Siokas, V., Martzoukou, M., Zoupa, E., Messinis, L., et al. (2023). Computer-Based Cognitive Training vs. In Paper-and-pencil training for language and cognitive deficits in Greek patients with mild Alzheimer's disease: A preliminary study. Healthcare (, Vol. 11, p. 443). Basel, Switzerland: 10.3390/healthcare11030443. 36767018 PMC9914594

[ref37] Gollan, T. H., Garcia, D. L., Murillo, M., Vargas, J., Pulido, B., & Salmon, D. P. (2023). Sprinting in two languages: Picture naming performance of older Spanish-English bilinguals on the multilingual naming test Sprint 2.0. Neuropsychology, 38, 653–664. 10.1037/neu0000958. PMC12930705 38990683

[ref38] Gollan, T. H., Garcia, D. L., Stasenko, A., Murillo, M., Kim, C., Galasko, D., et al. (2024). The MINT Sprint 2.0: A picture naming test for detection of naming impairments in Alzheimer's disease and in preclinical AD. Alzheimers Dement, 20, 112–123. 10.1002/alz.13381. 37464962 PMC10916946

[ref39] Grammatikopoulos, I. A., Sinoff, G., Alegakis, A., Kounalakis, D., Antonopoulou, M., & Lionis, C. (2010). The short anxiety screening test in Greek: Translation and validation. Ann General Psychiatry, 9, 1. 10.1186/1744-859X-9-1. PMC2819236 20051118

[ref41] Grothe, M. J., Moscoso, A., Ashton, N. J., Karikari, T. K., Lantero-Rodriguez, J., Snellman, A., et al. (2021). Associations of fully automated CSF and novel plasma biomarkers with Alzheimer disease neuropathology at autopsy. Neurology, 97, e1229–e1242. 10.1212/WNL.0000000000012513. 34266917 PMC8480485

[ref42] Hampel, H., O'Bryant, S. E., Molinuevo, J. L., Zetterberg, H., Masters, C. L., Lista, S., et al. (2018). Blood-based biomarkers for Alzheimer disease: Mapping the road to the clinic. Nat Rev Neurol, 14, 639–652. 10.1038/s41582-018-0079-7. 30297701 PMC6211654

[ref43] Hamrick, P., Sanborn, V., Ostrand, R., & Gunstad, J. (2023). Lexical speech features of spontaneous speech in older persons with and without cognitive impairment: Reliability analysis. JMIR Aging, 6, e46483. 10.2196/46483. 37819025 PMC10583496

[ref44] Hsieh, S., Schubert, S., Hoon, C., Mioshi, E., & Hodges, J. R. (2013). Validation of the Addenbrooke's cognitive examination III in frontotemporal dementia and Alzheimer's disease. Dement Geriatr Cogn Disord, 36, 242–250. 10.1159/000351671. 23949210

[ref45] Hudson, T. E., Conway, J., Rizzo, J.-R., Martone, J., Chou, L. T., Balcer, L. J., et al. (2022). Rapid automatized picture naming in an outpatient concussion center: Quantitative eye movements during the mobile universal lexicon evaluation system (MULES) test. Clinical and Translational Neuroscience, 6, 18. 10.3390/ctn6030018.

[ref46] Ingala, S., Tomassen, J., Collij, L. E., Prent, N., van 't Ent, D., Ten Kate, M., et al. (2021). Amyloid-driven disruption of default mode network connectivity in cognitively healthy individuals. Brain communications, 3, fcab201. 10.1093/braincomms/fcab201. 34617016 PMC8490784

[ref47] Jack, C. R., Jr., Andrews, J. S., Beach, T. G., Buracchio, T., Dunn, B., Graf, A., et al. (2024). Revised criteria for diagnosis and staging of Alzheimer's disease: Alzheimer's Association workgroup. Alzheimers Dement, 20, 5143–5169. 10.1002/alz.13859. 38934362 PMC11350039

[ref48] Jiang, Q., Liu, J., Huang, S., Chen, Y., Zhang, L., & Wang, X. (2025). Antiageing strategy for neurodegenerative diseases: From mechanisms to clinical advances. Signal transduction and targeted. Therapy, 10, 76. 10.1038/s41392-025-02145-7. PMC11891338 40059211

[ref49] Jiang, Y., & An, X. (2025). Speech differences between aged women with and without early Alzheimer's disease: Linguistic indicators of cognitive decline. Acta Psychol, 256, 105008. 10.1016/j.actpsy.2025.105008. 40233652

[ref50] Kaplan, E., Goodglass, H., & Weintraub, S. (1983) Boston naming test (BNT) [database record]. 102, APA PsycTests. 10.1037/t27208-000.

[ref51] Karalı, F. S., Tosun, S., Eskioğlu, E. İ., Çınar, N., & Macoir, J. (2025). The TDQ-60 tr-a picture-naming test to assess anomia in Turkish adults and the elderly: Normative data and validation study in Alzheimer's disease and mild cognitive impairment. Archives of clinical neuropsychology : the official journal of the National Academy of Neuropsychologists, 40, 529–540. 10.1093/arclin/acaf005. 39881515

[ref52] Kim, H., & Choi, H. (2024). The relationship of word-finding behaviors and naming in mild cognitive impairment and dementia of Alzheimer’s type. Communication Sciences & Disorders, 29, 618–630. 10.12963/csd.240047.

[ref53] Kitaigorodsky, M., Curiel Cid, R. E., Crocco, E., Gorman, K. L., González-Jiménez, C. J., Greig-Custo, M., et al. (2021). Changes in LASSI-L performance over time among older adults with amnestic MCI and amyloid positivity: A preliminary study. J Psychiatr Res, 143, 98–105. 10.1016/j.jpsychires.2021.08.033. 34464879 PMC8557121

[ref54] Kounti, F., Tsolaki, M., & Kiosseoglou, G. (2006). Functional cognitive assessment scale (FUCAS): A new scale to assess executive cognitive function in daily life activities in patients with dementia and mild cognitive impairment. Hum Psychopharmacol, 21, 305–311. 10.1002/hup.772. 16856217

[ref55] Lemprière, S. (2023). Neuroinflammation, not amyloid-β deposition, associated with brain network dysfunction in AD. Nat Rev Neurol, 19, 66. 10.1038/s41582-022-00770-2. 36609713

[ref56] Lin, R. R., Xue, Y. Y., Li, X. Y., Chen, Y. H., Tao, Q. Q., & Wu, Z. Y. (2021). Optimal combinations of AT(N) biomarkers to determine longitudinal cognition in the Alzheimer's disease. Front Aging Neurosci, 13, 718959. 10.3389/fnagi.2021.718959. 34421579 PMC8377373

[ref57] Macoir, J., Landry, M., & Hudon, C. (2025). Normative data for the famous people fluency test in the adult French-Quebec population and validation study in mild cognitive impairment and Alzheimer's disease. Archives of clinical neuropsychology : the official journal of the National Academy of Neuropsychologists, 40, 708–717. 10.1093/arclin/acae053. 39004918 PMC12079421

[ref58] Martínez-Ferreiro, S. (2024). Naming as a window to word retrieval changes in healthy and pathological ageing: Methodological considerations. Int J Lang Commun Disord, 59, 68–83. 10.1111/1460-6984.12827. 36507588 PMC13507608

[ref59] McAleese, K. E., Walker, L., Erskine, D., Thomas, A. J., McKeith, I. G., & Attems, J. (2017). TDP-43 pathology in Alzheimer's disease, dementia with Lewy bodies and ageing. Brain Pathol, 27, 472–479. 10.1111/bpa.12424. 27495267 PMC8029292

[ref60] Montine, T. J., Cholerton, B. A., Corrada, M. M., Edland, S. D., Flanagan, M. E., Hemmy, L. S., et al. (2019). Concepts for brain aging: Resistance, resilience, reserve, and compensation. Alzheimers Res Ther, 11, 22. 10.1186/s13195-019-0479-y. 30857563 PMC6410486

[ref61] Morkovina, O., Manukyan, P., & Sharapkova, A. (2024). Picture naming test through the prism of cognitive neuroscience and linguistics: Adapting the test for cerebellar tumor survivors-or pouring new wine in old sacks? Front Psychol, 15, 1332391. 10.3389/fpsyg.2024.1332391. 38566942 PMC10985186

[ref62] Mueller, K. D. (2023). Using discourse as a measure of early cognitive decline associated with Alzheimer’s disease biomarkers. In Kong, A. P. H. (Ed.), Spoken discourse impairments in the neurogenic populations (pp. [page range if known]). Cham: 10.1007/978-3-031-45190-4_4 Springer.

[ref63] Nasreddine, Z. S., Phillips, N. A., Bédirian, V., Charbonneau, S., Whitehead, V., Collin, I., et al. (2005). Montreal cognitive assessment (MoCA) [database record]. APA PsycTests, 53, 693–699. 10.1037/t27279-000. 15817019

[ref64] Nichols, E., Szoeke, C. E., Vollset, S. E., Abbasi, N., Schutte, C., & Murray, C. J. (2022). Estimation of the global prevalence of dementia in 2019 and forecasted prevalence in 2050: An analysis for the global burden of disease study 2019. Lancet Public Health, 7, e105–e125. 10.1016/S2468-2667(21)00249-8. 34998485 PMC8810394

[ref65] Paplikar, A., Varghese, F., Alladi, S., Vandana, V. P., Darshini, K. J., Iyer, G. K., et al. (2022). Picture-naming test for a linguistically diverse population with cognitive impairment and dementia. Int J Lang Commun Disord, 57, 881–894. 10.1111/1460-6984.12728. 35522006

[ref66] Patricacou, A., Psallida, E., Pring, T., & Dipper, L. (2007). The Boston naming test in Greek: Normative data and the effects of age and education on naming. Aphasiology, 21, 1157–1170. 10.1080/02687030600670643.

[ref67] Petersen, R. C. (2004). Mild cognitive impairment as a diagnostic entity. J Intern Med, 256, 183–194. 10.1111/j.1365-2796.2004.01388.x. 15324362

[ref67a] Petersen, R. C. (2016) Mild Cognitive Impairment. Continuum, 22, 404–418. 10.1212/con.0000000000000313. 27042901 PMC5390929

[ref68] Poptsi, E., Moraitou, D., Eleftheriou, M., Kounti-Zafeiropoulou, F., Papasozomenou, C., Agogiatou, C., et al. (2019). Normative data for the Montreal cognitive assessment in Greek older adults with subjective cognitive decline, mild cognitive impairment and dementia. J Geriatr Psychiatry Neurol, 32, 265–274. 10.1177/0891988719853046. 31159629

[ref70] Poptsi, E., Moraitou, D., Tsardoulias, E., Symeonidis, A. L., Papaliagkas, V., & Tsolaki, M. (2023). R4Alz-revised: A tool able to strongly discriminate 'subjective cognitive decline' from healthy cognition and ‘minor neurocognitive disorder’. Diagnostics (Basel, Switzerland), 13, 338. 10.3390/diagnostics13030338. 36766444 PMC9914647

[ref71] Porsteinsson, A. P., Isaacson, R. S., Knox, S., Sabbagh, M. N., & Rubino, I. (2021). Diagnosis of early Alzheimer's disease: Clinical practice in 2021. J Prev Alzheimers Dis, 8, 371–386. 10.14283/jpad.2021.23. 34101796 PMC12280795

[ref72] Pournaghdali, A., Schwartz, B. L., & Soto, F. A. (2025). Tip-of-the-tongue and feeling-of-knowing experiences enhance metacognitive sensitivity of confidence evaluation of semantic memory. J Cogn, 8, 33. 10.5334/joc.442. 40322618 PMC12047626

[ref73] Rami, L., Mollica, M. A., García-Sánchez, C., Saldaña, J., Sanchez, B., Sala, I., et al. (2014). The subjective cognitive decline questionnaire (SCD-Q): A validation study. J Alzheimers Dis, 41, 453–466. 10.3233/JAD-132027. 24625794

[ref74] Reisberg, B., Torossian, C., Shulman, M. B., Monteiro, I., Boksay, I., Golomb, J., et al. (2019). Two year outcomes, cognitive and Behavioral markers of decline in healthy, cognitively normal older persons with global deterioration scale stage 2 (subjective cognitive decline with impairment). Journal of Alzheimer's disease : JAD, 67, 685–705. 10.3233/JAD-180341. 30689585

[ref75] Roberts, R., & Knopman, D. S. (2013). Classification and epidemiology of MCI. Clin Geriatr Med, 29, 753–772. 10.1016/j.cger.2013.07.003. 24094295 PMC3821397

[ref76] Sewell, K. R., Oberlin, L. E., Karikari, T. K., Olvera-Rojas, M., Wan, L., Morris, J. K., et al. (2025). Blood biomarkers differentiate AD-related versus non-AD-related cognitive deficits. Alzheimers Dement, 21, e14619. 10.1002/alz.14619. 40110626 PMC11923558

[ref77] Shaikh, K. T., Zaidi, K. B., Wong Gonzalez, D., Dimech, C., Gilson, Z. M., Stokes, K. A., et al. (2025). Cultural bias in the assessment of language: A closer look at the Boston naming test among multicultural Canadian older adults. Appl Neuropsychol Adult, 1–13. Advance online publication, 1–13. 10.1080/23279095.2024.2449172. 39787017

[ref78] Smith, C., Ivanova, I., & Fernandez, L. (2022). Naming tests in neuropsychological assessment: A review of applications and methods. Cogn Sci, 3, 210–230. 10.1016/j.jcogsci.2021.210033.

[ref79] Soncu Büyükişcan, E. (2025). Neuropsychology of Alzheimer’s disease: From preclinical phase to dementia. Appl Neuropsychol Adult, 1–9, 1–9. 10.1080/23279095.2025.2469236. 39982692

[ref80] Stasenko, A., Jacobs, D. M., Salmon, D. P., & Gollan, T. H. (2019). The multilingual naming test (MINT) as a measure of picture naming ability in Alzheimer's disease. Journal of the International Neuropsychological Society : JINS, 25, 821–833. 10.1017/S1355617719000560. 31248465 PMC6757330

[ref81] Stiver, J., Staffaroni, A. M., Walters, S. M., You, M. Y., Casaletto, K. B., Erlhoff, S. J., et al. (2022). The rapid naming test: Development and initial validation in typically aging adults. Clin Neuropsychol, 36, 1822–1843. 10.1080/13854046.2021.1900399. 33771087 PMC8464629

[ref81a] Szymkowicz, S. M., Gerlach, A. R., Homiack, D., & Taylor, W. D. (2023) Biological factors influencing depression in later life: role of aging processes and treatment implications. Translational Psychiatry, 13. 10.1038/s41398-023-02464-9. PMC10169845 37160884

[ref82] Teylan, M., Mock, C., Gauthreaux, K., Chen, Y. C., Chan, K. C. G., Hassenstab, J., et al. (2020). Cognitive trajectory in mild cognitive impairment due to primary age-related tauopathy. Brain J Neurol, 143, 611–621. 10.1093/brain/awz403. PMC7009602 31942622

[ref83] Tsatali, M., Moraitou, D., Gialaouzidis, M., Bakoglidou, E., Psaltis, V., Bertzes, N., et al. (2024). Discriminant potential of the Alzheimer's disease assessment scale-cognitive subscale (ADAS-cog) in Greek older adults with subjective cognitive decline and mild cognitive impairment. Journal of Alzheimer's disease reports, 8, 543–554. 10.3233/ADR-230151. PMC10977457 38549629

[ref84] Vergallo, A., Mégret, L., Lista, S., Cavedo, E., Zetterberg, H., Blennow, K., et al. (2019). Plasma amyloid β 40/42 ratio predicts cerebral amyloidosis in cognitively normal individuals at risk for Alzheimer's disease. Alzheimers Dement, 15, 764–775. 10.1016/j.jalz.2019.03.009. 31113759

[ref84a] Wang, M.-Y., Chen, K.-L., Huang, Y.-Y., Chen, S.-F., Wang, R.-Z., Zhang, Y., et al. (2025) Clinical utility of cerebrospinal fluid Alzheimer’s disease biomarkers in the diagnostic workup of complex patients with cognitive impairment. Translational Psychiatry, 15. 10.1038/s41398-025-03345-z. PMC11976989 40195333

[ref85] Weinstein, A. M., Gujral, S., Butters, M. A., Bowie, C. R., Fischer, C. E., Flint, A. J., et al. (2022). Diagnostic precision in the detection of mild cognitive impairment: A comparison of two approaches. The American journal of geriatric psychiatry : official journal of the American Association for Geriatric Psychiatry, 30, 54–64. 10.1016/j.jagp.2021.04.004. 34023224 PMC8720569

[ref86] World Medical Association (2013). World medical association declaration of Helsinki: Ethical principles for medical research involving human subjects. JAMA, 310, 2191–2194. 10.1001/jama.2013.281053. 24141714

[ref87] Yoon, K. H., Moon, Y. S., & Kim, D. H. (2023). The impact of depression on language function in individuals with Alzheimer’s disease: A pre/post-treatment design. Ann General Psychiatry, 22, 4. 10.1186/s12991-023-00433-6. PMC9898976 36737766

[ref88] Yu, X., Shi, R., Zhou, X., Zhang, M., Cai, Y., Jiang, J., et al. (2024). Correlations between plasma markers and brain aβ deposition across the AD continuum: Evidence from SILCODE. Alzheimers Dement, 20, 6170–6182. 10.1002/alz.14084. 38982860 PMC11497764

[ref89] Zhang, H., Jiang, X., Ma, L., Wei, W., Li, Z., Chang, S., et al. (2022). Role of aβ in Alzheimer's-related synaptic dysfunction. Front Cell Dev Biol, 10, 964075. 10.3389/fcell.2022.964075. 36092715 PMC9459380

[ref90] Zhang, J., Zhang, Y., Wang, J., Xia, Y., Zhang, J., Chen, L., et al. (2024). Recent advances in Alzheimer’s disease: Mechanisms, clinical trials and new drug development strategies. Signal Transduct Target Ther, 9, 211. 10.1038/s41392-024-01911-3. 39174535 PMC11344989

[ref91] Zhu, D., Montagne, A., & Zhao, Z. (2021). Alzheimer's pathogenic mechanisms and underlying sex difference. Cellular and molecular life sciences : CMLS, 78, 4907–4920. 10.1007/s00018-021-03830-w. 33844047 PMC8720296

[ref92] Zimmerer, V. C., Weiner, K., & Varley, R. (2021). Mobile universal lexicon evaluation system (MULES): A validated tool for multilingual cognitive assessment. Clin Neuropsychol, 23, 63–78. 10.1080/13854046.2020.1812558.

